# Perceptions of young Jordanian adults to proposed anti-tobacco pictorial warning labels

**DOI:** 10.1186/1471-2458-11-414

**Published:** 2011-05-31

**Authors:** Feras I Hawari, Rasha K Bader, Hamza M Beano, Nour A Obeidat, Hiba S Ayub, Malek A Habashneh, Aisha S Shtaiwi, Rawan A Shihab, Hala N Madanat, Thomas E Novotny

**Affiliations:** 1 Cancer Control Office, King Hussein Cancer Center, Amman, Jordan; 2 Faculty of Medicine, University of Jordan, Amman, Jordan; 3 Health Awareness and Promotion Directorate, Jordanian Ministry of Health, Jordan; 4 Graduate School of Public Health, San Diego State University, San Diego, CA, USA; 5 San Diego Prevention Research Center, San Diego, CA, USA; 6 Family and Preventive Medicine, University of California San Diego, San Diego, CA, USA

**Keywords:** health warnings, Middle-East, salience, fear-elicitation, tobacco

## Abstract

**Background:**

In commitment to the Framework Convention on Tobacco Control (FCTC), four new pictorial warnings are now being proposed for display on cigarette packages sold in Jordan. The aim of this study was to gauge the immediate perceptions of young Jordanian adults towards these new pictorials and compare these perceptions to those of the pictorial currently being used in the country.

**Methods:**

A cross-sectional survey was conducted on a convenience sample of youth aged 17-26. The interviewer-administered survey gauged participants' perceptions of salience, fear elicitation, and gained information as well as participants' motivation to remain non-smokers or quit smoking after viewing each of the four proposed new pictorials as well as the current pictorial used in Jordan. Perceptions regarding each new pictorial were compared to the current pictorial.

**Results:**

A total of 450 surveys were included in the analysis. The sample (mean age 20.9) was 51.6% female and 31.3% cigarette (regular or occasional) smokers. In smokers, only one proposed pictorial had significantly more smokers perceiving it as salient or adding to information when compared to the current pictorial. More smokers reported fear when observing the proposed pictorials compared with current pictorial, but overall proportions reporting fear were generally less than 50%. Furthermore, all new pictorials motivated significantly more smokers to consider quitting compared with the current pictorial; however, the overall proportion of smokers reporting motivation was < 25%. Among nonsmokers, significantly more respondents perceived the new pictorials as salient and fear-eliciting compared to the old pictorial, but there were no major differences in information added. Motivation to remain non-smokers was comparable between the old and new pictorials.

**Conclusion:**

Given the variability of response across both smokers and nonsmokers, and across the three elements of perception (salience, added information, fear) for each pictorial, further testing of the pictorials in a more diverse sample of Jordanian young adults prior to launch is recommended.

## Background

Pictorial warnings on cigarette packages have been identified as important and cost effective health communication strategies [1-4]. They communicate information regarding the health risks associated with cigarette smoking [2,5-7], support individuals intentions to quit or not to initiate smoking [8-14], and have been shown to increase smoking cessation rates [9,15,16].

Article 11 of the Framework Convention on Tobacco Control (FCTC), the first international public health treaty, provides important guidelines regarding health warnings on cigarette packages [17]. Consistent with research indicating greater effectiveness of warnings combining both pictures and text compared to text-only warnings [18-20], the FCTC guidelines emphasize the need for the health warnings on cigarette packages to contain both text and pictures [17]. The guidelines also stress that pictorial warnings should cover no less than 30 percent, but preferably at least 50 percent of the cigarette package [17]. Additional guidelines emphasize the need to place these warnings on the front and back of packages; use color; rotate two different sets of health warnings; include a variety of messages covering "advice on cessation, the addictive nature of tobacco, adverse economic and social outcomes, and the impact of tobacco use on significant others"; use multiple languages in places where different languages are used; and use pictorials which are more graphic and include shocking images.

**Figure 1:**
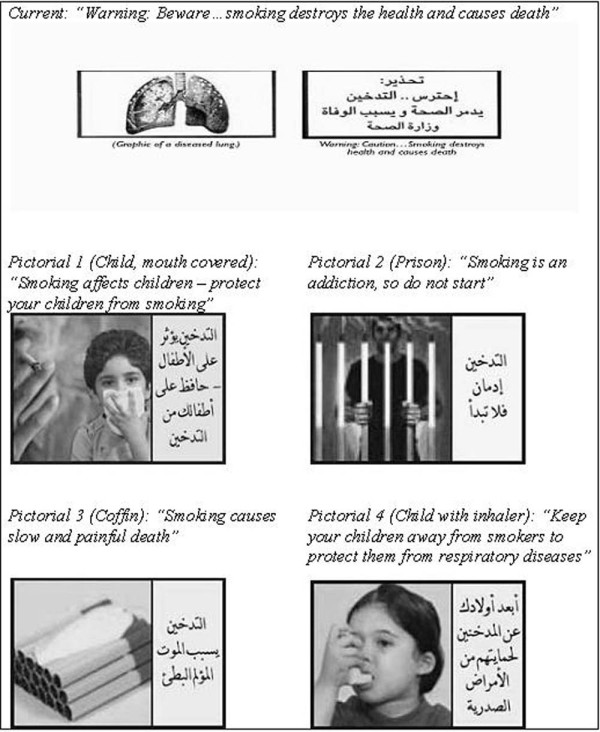
Pictorial cigarette package warnings, Jordan

Countries vary considerably in their mandate and in how they select their pictorial warnings. Jordan ratified the FCTC in 2004 [21], and the requirement for having pictorial warnings on cigarette packs went into effect in early 2006, making it the first country in the region to display warnings on cigarette packs (followed in 2008 by Egypt, which now enforces the use of four rotating warning labels) [22,23]. One pictorial was approved in Jordan during the FCTC ratification period and remains in use. The pictorial consists of a warning text on one side of the cigarette pack and a pictorial warning on the other side covering 33% of the principle display area of the tobacco package [22]. To date, no data exist on the effectiveness of this warning on the population. To enhance compliance with FCTC guidelines, and based on evidence suggesting that larger, more contrasting warnings have a greater impact on public perceptions [24], Jordan is now considering changing to four new pictorial warnings, each covering about 40% of the package display area (Figure 1).

The move towards more visually prominent anti-tobacco messages is important given the magnitude of the smoking problem in the country. The overall prevalence of smoking in Jordan is approximately 28% among adults, but is particularly high among males. For example, among younger adult males aged 18 to 24, the prevalence of smoking has been estimated at 42.2% and becomes higher (62.7%) among 25 to 34 year-old men [25]. Thus, plans to enhance pictorial warnings is a substantial and positive move in Jordan's tobacco control efforts, and establishes it as one of the first countries in the Middle East to use a selection of pictorial health warnings. It is also likely that Jordan's experience may prove valuable to similarly structured developing countries with comparable cultures. However, the pictorials currently proposed for use in Jordan are not as graphic as those used in other parts of the world. Thus, they deviate from the current global consensus that graphic and often shocking images are considered to have a greater impact [2,8-10,15,16,19,20]. Careful testing of such warnings is warranted before implementation [1,17], especially if these warnings deviate from the evidence-based international consensus.

It is therefore useful to study the potential effect of these pictorials prior to launching them into the Jordanian market. Initial evidence provided by such a study can be a gauge of how these pictorials will be perceived, and study results can be used to advise regulators on possible modifications to improve their effectiveness. This is particularly needed in Jordan, where the process of implementing such pictorials is time-consuming and difficult to reverse. The impact of using these pictorials must be carefully anticipated in order to increase the chances of the health warnings promoting positive health behavior change.

The purpose of this study was to gauge the immediate effect of the four proposed pictorial warnings on a sample of young Jordanian adults. Comparisons were made between the proposed pictorial warnings and the existing pictorial warnings regarding perceptions of salience, fear elicitation and gained information. In addition, comparisons were made regarding the participants' motivation to remain non-smokers or to quit smoking after viewing each of the pictorials.

## Methods

### Sample

The cross-sectional convenience sample for this study was obtained by recruiting young adults aged 17-26 years in the community (more than 95% of the sample was composed of college students). The choice of this age group was based on previous data suggesting high rates of smoking initiation during college years in Jordan [26] and the high rate of effectiveness of pictorial warnings in providing this age group with information and making cigarettes less attractive [4].

### Instrument

The instrument used for the study was an adaptation of an Arabic survey (see additional files 1 and 2) originally developed by the Department of Health Promotion and Community Health, at the School of Health Sciences at the American University of Beirut (Personal Communication, July 17, 2010). The survey consisted of three sections.

The first section (see additional files 1 and 2) asked about smoking behavior and opinions of respondents on the harms of smoking.

The second section (see additional files 1 and 2) assessed the impact of each of the four proposed warning pictorials as well as the one currently on the market on 1) participant's perceptions of salience, fear elicited and gained information after viewing each pictorial and 2) his/her motivation to not initiate smoking (if a non-smoker) or to quit smoking (if a smoker) after viewing each pictorial. For each pictorial, perceptions of salience (ranging from "not noticeable to "noticeable and attracts attention"), extent of fear elicited (ranging from "not scary" to "very scary"), and degree of information added (ranging from "not informative" to "informative and adds to my knowledge") were assessed through a five-point Likert scale, with '1' indicating the weakest perception, and '5' indicating the strongest perception. Motivation to quit smoking ("seeing this warning motivates me to try to quit smoking") or remain a nonsmoker ("seeing this warning motivates me to remain a non smoker") was also measured using a five-point Likert scale ranging from strong disagreement to strong agreement to engage in a positive behavior. Each pictorial was shown to the respondent separately, and all questions regarding perceptions and motivation were repeated for each pictorial. Respondents were also asked one open-ended question regarding any comments they might have about the pictorial warning they had just seen.

The third section (see additional files 1 and 2) consisted of three basic demographic questions: 1) age, 2) gender, and 3) level of education.

The instrument is available electronically through BMC.

### Procedures

The Institutional Review Board at the King Hussein Cancer Center approved the study prior to data collection (the research was deemed minimal risk and written informed consent was waived).

The surveyors group for the study was composed of 32 volunteer medical students from three Jordanian universities (18 were female; and three of the 32 smoked). Surveyors were split into two groups and received two-hour training by the principal investigator on the purpose of the study, the instrument content, and the data collection methods. In addition, surveyors received a detailed sheet with instructions about how to approach respondents and information to provide regarding the study. Surveyors were then asked to recruit participants from the community, with most recruitment planned to take place on the campuses of several Jordanian universities. Once a potential participant was approached, the purpose of the study was explained and participants were told that their participation was voluntary, that they could withdraw at any time, and that no identifying information would be collected. An oral consent was obtained from participants prior to survey administration and recorded by the surveyors. The survey took approximately 30 minutes to complete.

### Data Analyses

Basic univariate and bivariate analyses were performed, and responses for each pictorial were compared to the responses observed for the current pictorial. Responses to five-point scales were dichotomized, whereby, for perceptions or motivation, responses of '4' and '5' were considered positive and '1', '2', and '3' were considered negative. For example, a smoking respondent assigning a '4' or '5' (on the five point scale for salience) and '1', '2' or '3' (on the five point scale for motivation) for a pictorial was analyzed as a respondent perceiving the pictorial to be salient (can attract attention), but not being a motivator to quit smoking.

For each of the four new pictorial warnings, proportions of respondents ranking a pictorial on perception and motivation scales were compared with reported proportions for the existing pictorial using the Chi-square statistics. Analyses were stratified by smoking status, and comparisons were considered significant at p < 0.05.

## Results

Out of 564 subjects approached by surveyors, a total of 478 subjects agreed to participate in the survey. Due to poor quality of data entry in a small selection of returned surveys, a final number of 450 completed surveys were used in the final analysis, representing an approximate response rate of 80%. The mean age of respondents was 20.9 (SD = 1.65), and males and females were roughly equally distributed in the sample (Table 1). The proportion of regular or occasional cigarette smokers was 31.3%, the majority (78.7%) of whom was male. The majority of respondents acknowledged that smoking was harmful to both smokers and nonsmokers. The majority of respondents also reported having previously seen the existing pictorial warning.

**Table 1:** Demographics, familiarity with pictorial warnings on cigarette packages, and beliefs about smoking by smoking status, adults aged 17-26 years in Jordan

Characteristic	Non-smokers (N = 309)	Smokers (N = 141)	Overall (N = 450)
**Mean age (range)**	20.7 (SD = 1.64)	21.24 (SD = 1.64)	20.9 (SD = 1.65)

**Gender (% males)**	121 (39.2%)	111 (78.7%)	232 (48.4%)

**Familiarity with current pictorial (previously seen)**	294 (95.1%)	139 (98.6%)	433 (96.2%)

**Prior beliefs**			
Smoking is harmful to smokers	303 (98.1%)	129 (91.5%)	432 (96%)
Smoking is harmful to both smokers and nonsmokers	304 (98.3%)	125 (88.6%)	429 (95.3%)

**Figure 2:**
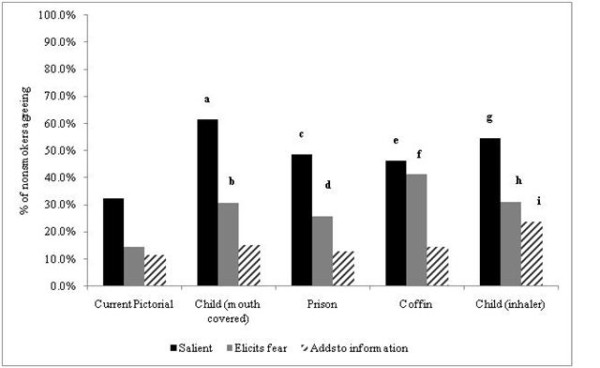
**Non-smokers' perceptions of salience, fear elicitation, and gaining of information for each of four new and one current pictorial warnings, adults aged 17-26 years, Jordan, 2010**. *a to i - Significantly greater proportions than current pictorial: a, (61.5% vs. 32.4%, p < 0.0001); b, (30.7% vs. 14.6%, p < 0.0001);c, (48.5% vs. 32.4%, p < 0.001); d, (25.9% vs. 14.6%, p = 0.005); e,(46.3% vs. 32.4%, p = 0.0004); f, (41.4% vs. 14.6%, p < 0.0001); g, (54.7% vs. 32.4%, p < 0.0001); h, (31.1% vs. 14.6%, p < 0.0001); i, (23.9% vs. 11.7%, p < 0.0001)*.

Non-smokers reported significantly more frequently each of the four new pictorial warnings as salient and eliciting fear compared to the existing pictorial warning. However, only one proposed pictorial warning (child using inhaler) provided information to significantly more non-smokers than the existing pictorial did (23.95% versus 11.7%, p < 0.0001, Figure 2).

**Figure 3:**
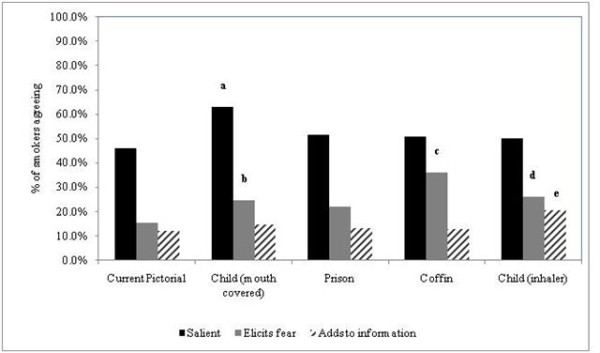
**Smokers' perceptions of salience, fear elicitation, and gaining of information for each of four new and one current pictorial warnings, adults aged 17-26 years, Jordan, 2010**. *a to e - Significantly greater proportions than current pictorial: a, (63.1% vs. 46.1%, p = 0.004); b,(24.8% vs. 15.6%, p = 0.05); c, (36.2% vs. 15.6%, p < 0.0001); d, (26.2% vs. 15.6%, p = 0.03); e, (20.6% vs. 12.1%, p = 0.05)*.

In smokers, only one of the proposed pictorial warnings (child covering mouth) had significantly more respondents perceiving the pictorial warning as salient compared with the current pictorial warning (63.1% versus 46.1%, p = 0.004, Figure 3). As was the case with non-smokers, only the pictorial warning representing the child using an inhaler had significantly more respondents perceiving the new pictorial as adding to their information about the health risks of smoking when compared with the current pictorial warning (20.6% versus 12.1%, p = 0.05, Figure 3). Regarding perceptions of fear elicitation, only one pictorial warning (prison) did not have a significant effect on fear-elicitation. Among the remaining pictorials that did, that with a coffin had the most substantial fear-eliciting effect (although there were no differences in salience and information gained relative to the current pictorial (Figure 3).

Among both smokers and nonsmokers, no more than 42% of respondents perceived any of the proposed pictorials as fear-eliciting, and no more than 25% of respondents perceived any of the proposed pictorials as adding to their information.

**Figure 4:**
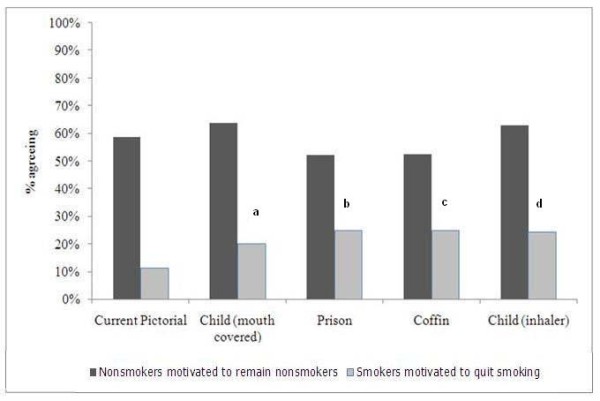
**Motivation to quit cigarette smoking or remain nonsmoker after viewing current and new pictorial package warnings, adults aged 17-26 years, Jordan, 2010**. *a to d - Significantly greater proportions than current pictorial: a, (19.9% vs. 11.3%, p = 0.05); b, (24.8% vs. 11.3% p = 0.003); c, (24.8% vs. 11.3%, p = 0.003); d, (24.1% vs. 11.3%, p = 0.005)*.

With regards to motivation to remain engaged in a positive behavior (refrain from smoking) among nonsmokers, no significant differences were detected when comparing each of the four new pictorials with the current warning. Among smokers, all new pictorial warnings motivated significantly more respondents to consider quitting than the current warning. However, no more than 30% of smokers reported motivation to quit (Figure 4).

## Discussion

To the authors' knowledge, there are no published evaluations of pictorial warnings in the Middle East. Given that neighboring countries are likely to embark on similar tobacco control initiatives, our results can provide insights to other tobacco control authorities in the region. For example, one of the proposed pictorials (child covering mouth) to be used in Jordan is currently being used in Egypt.

We compared four proposed pictorials to a pictorial that has been in the market for several years and whose effects have likely been exhausted. Thus, we anticipated that any new pictorial would (upon first observation) likely be perceived as more salient, adding more information, or eliciting more fear, than the previous pictorial (our baseline).

Among smokers, for most of the proposed pictorials (with the exception of the child covering mouth), the number of respondents reporting salience was comparable to the old pictorial. With regard to fear-elicitation, most respondents perceived the proposed warnings (with the exception of smoking as a prison) as fear-eliciting. Although this is a positive indication of their efficacy, it is important to point out that fear-elicitation was only reported by less than half (42%) of smoking respondents for all of the pictorials. In addition, despite being new (relative to the current pictorial), with the exception of one proposed warning (child with inhaler), perception of added information for the proposed pictorials was comparable to that of the old warning. Furthermore, despite more reported motivation to quit smoking after viewing the new pictorials, the overall proportions reporting motivation to quit were generally low.

Among nonsmokers, respondents expressed higher perceptions of salience and fear elicitation when viewing the new pictorials compared with the existing warning. These results are consistent with previous research indicating that health warnings are more salient among non-smokers [1]. However, with the exception of one proposed warning (child with inhaler), perception of added information for the proposed pictorials was comparable to that of the old warning. Also, respondents reporting motivation not to initiate smoking were comparable after viewing the new warnings and the old pictorial. These results imply that if stronger motivation or added information among nonsmokers is desired from these new pictorials, they may need to be revised.

Our data suggest that the proposed pictorials may not trigger sufficient perceptions of salience (particularly for smokers) and added information for either smokers or nonsmokers. Thus, Jordan may benefit from specifically introducing more graphic and informative pictorials, in line with the general international consensus. Previous research suggests combining graphic warnings with supportive cessation information (which was not available in any of the proposed warnings) [1]. Jordan also might benefit from selecting a larger group of pictures to address various specific elements of perception, given that it is difficult to capture all elements in one pictorial warning and that the effects of fewer pictorials can be quickly exhausted [17]. Furthermore, the variability in response across pictorials and by smoking status emphasizes the need for carefully selecting and using various pictorials that can resonate across a diverse audience, since the intended audience will vary in age, levels of literacy, socioeconomic status, and smoking status. For example, in our study, some respondents indicated that messages containing children did not sufficiently express the dangers of smoking to smokers, and did not seem relevant to young adults who do not have children. FCTC guidelines also have pointed to the need for careful consideration of literacy when choosing the pictorial warnings [17].

Finally, with regards to activities that could improve public perception regarding tobacco, supplementary educational campaigns can be useful, particularly after observing the low proportion of respondents reporting added knowledge after viewing the pictorials and text. Such campaigns can also address waterpipes, given that the latter are a common form of tobacco with fewer control measures (the proposed pictorial warnings only apply to cigarette packages). It is also recommended that Jordan strengthens its document research on the tobacco industry in order to gather information regarding the messages being sent and the groups being targeted in the country by tobacco industry advertising. Accordingly, the Ministry of Health can ensure that the information conveyed by the pictorial warnings counters these messages effectively. Document research in other countries has provided important information to strengthen the impact of the tobacco control policies on smoking initiation and cessation [27].

Our study had some limitations: we used a convenience sample of youth, which is not representative of the final target audience for the proposed pictorials. Thus, similar surveys of other demographic groups can better inform decision-makers of the usefulness of the warnings. The study is also cross-sectional in design and does not capture temporal changes that may occur after prolonged observation of the pictorial warnings. Population-based monitoring over time would be needed to better understand the impact of warnings. Nevertheless, more provocative pictorials may have elicited stronger responses than those observed in our sample.

## Conclusion

Our study presents a first step toward understanding local perceptions and efficacy of tobacco package health warnings, and our selected group represents a critical and populous segment of the Jordanian population (youth) that is at high likelihood of smoking or beginning to smoke. While more research is recommended, our results point to some factors that, if addressed, could improve the impact of new pictorial warnings to be launched in Jordan.

## Competing interests

The authors declare that they have no competing interests.

## Authors' contributions

FH contributed to study design, data interpretation, and manuscript writing and review; RB contributed to study design, data analysis and interpretation, and manuscript writing and review; HB contributed to data collection and management, and manuscript review; NO contributed to data analysis and interpretation, and manuscript writing and review; HA contributed to data interpretation and manuscript review; MH contributed to data acquisition and manuscript review; AS contributed to data collection and analysis; RS contributed to data interpretation and manuscript review; HM contributed to data interpretation, and manuscript writing and review; TN contributed to data interpretation and manuscript review.

All authors have read and approved the final manuscript.

## Pre-publication history

The pre-publication history for this paper can be accessed here:

http://www.biomedcentral.com/1471-2458/11/414/prepub

## Supplementary Material

Additional file 1 **Survey instrument - Arabic version**. This is the actual survey instrument that was utilized to collect data for this research. Click here for file

Additional file 2 **Survey instrument - English version**. This is a translation of the survey instrument that was utilized to collect data for this research. Click here for file
