# Supplementary material for: Perceptions of young Jordanian adults to proposed anti-tobacco pictorial warning labels
Source: BMC Public Health. 2011 May 31;11:414. doi: 10.1186/1471-2458-11-414 (PMC3141444; doi:10.1186/1471-2458-11-414)
Supplement: Additional file 1 — Survey instrument - Arabic version. This is the actual survey instrument that was utilized to collect data for this research. [file 1471-2458-11-414-S1.DOCX]

**تقييم أثر التحذيرات التصويرية على علب السجائر:**

**أراء ومعلومات عامة:**

| **إلى أي مدى توافق أو تعارض العبارات التالية:** | | أوافق بشدة | أوافق | محايد | أعارض | أعارض بشدة |
| --- | --- | --- | --- | --- | --- | --- |
| **B1** | التدخين يضرّ المدخنين |  |  |  |  |  |
| **B2** | التعرّض لدخان السجائر مضر للمدخنين وغير المدخنين |  |  |  |  |  |

| **يرجى وضع دائرة حول الإجابة الملائمة** | | | |
| --- | --- | --- | --- |
| **C1** | هل يوجد حاليًا أي تحذير صحي على علب السجائر؟ | 0 | لا **🡨 إنتقل إلى C4** |
|  |  | 1 | نعم |
| **C2** | أين موقعه؟ | 1 | على الجهة الأمامية |
|  |  | 2 | على الجهة الجانبية |
|  |  | 3 | على الجهة الخلفية |
|  |  | 4 | على الجهتين الأمامية والخلفية |
| **C3** | ماذا يقول؟ | 1 | التدخين إدمان ويمكن ان يضر بصحتك. |
|  |  | 2 | التدخين يضر بصحة الكبار والأطفال. |
|  |  | 3 | التدخين يدمر الصحة ويسبب الوفاة. |
|  |  | 4 | لا أعرف ما هو |
| **C4** | حاليًا أنت: | 1 | مدخن سجائر منتظم |
|  |  | 2 | مدخن سجائر في المناسبات فقط |
|  |  | 3 | مدخن سابق **🡨 إنتقل إلى D1** |
|  |  | 4 | غير مدخن **🡨 إنتقل إلى D1** |
| **C5** | هل تفكر بالإقلاع عن التدخين في ال 6 أشهر القادمة؟ | 0 | لا |
|  |  | 1 | نعم |
| **C6** | هل حاولت الإقلاع عن التدخين خلال ال12 شهرا الماضية؟ | 0 | لا **🡨 إنتقل إلى D1** |
|  |  | 1 | نعم |
| **C7** | ***متى كانت هذه المحاولة؟*** | 1 | منذ 6 أشهر أو أقل |
|  |  | 2 | منذ اكثر من 6 أشهر |

**ردود الفعل للتحذير الحالي:**

| **D1** | هل سبق أن رأيت هذا التحذير من قبل؟ | 0 | لا |
| --- | --- | --- | --- |
|  |  | 1 | نعم |

| **سوف تجد هنا مجموعة من الصفات. يرجى وضع دائرة حول الرقم الذي يصف ردة فعلك على التحذير الذي رأيته. مثلاً، إذا شعرت أنّه لم يكن ضعيفًا ولا قويًّا، قد تضع دائرة حول الرقم 3، ولكن إذا شعرت أنّه قويّ، قد تختار الرقم 4 أو 5.** | | | | | | | |
| --- | --- | --- | --- | --- | --- | --- | --- |
|  | أرى أن هذا التحذير | | | | | | |
| **D2** | **يمكن تجاهله بسهولة** | 1 | 2 | 3 | 4 | 5 | **مُلفِت** |
| **D3** | **ليس مخيفا أبدًا** | 1 | 2 | 3 | 4 | 5 | **مخيف جدًا** |
| **D4** | **لم يضف لمعلوماتي** | 1 | 2 | 3 | 4 | 5 | **أضاف لمعلوماتي** |

| **يرجى إكمال هذا القسم إذا كنتم من غير المدخنين فقط** | | أعارض بشدة | أعارض | محايد | أوافق | أوافق بشدة |
| --- | --- | --- | --- | --- | --- | --- |
| **D5** | **عند رؤيتي لهذا التحذير** إزدادت **رغبتي** بأن أبقى غير مدخّن |  |  |  |  |  |
| **D6** | **أثّر بي هذا التحذير حتى** أصبحت **واثق** مِن أنّني لن أبدأ بالتدخين |  |  |  |  |  |

| **يرجى إكمال هذا القسم إذا كنتم من المدخنين فقط** | | أعارض بشدة | أعارض | محايد | أوافق | أوافق بشدة |
| --- | --- | --- | --- | --- | --- | --- |
| **D7** | **أثّر بي هذا التحذير حتى** أنه **يدفعني** إلى الإقلاع عن التدخين |  |  |  |  |  |
| **D8** | **أثّر بي هذا التحذير حتى** أني **واثق** مِن أنّني أستطيع الإقلاع عن التدخين |  |  |  |  |  |
| **D9** | **هل لديك ملاحظات أخرى على التحذير؟** | | | | | |

**ردود الفعل للتحذير المقترح :**

| **سوف تجد هنا مجموعة من الصفات. يرجى وضع دائرة حول الرقم الذي يصف ردة فعلك على التحذير الذي رأيته. مثلاً، إذا شعرت أنّه لم يكن ضعيفًا ولا قويًّا، قد تضع دائرة حول الرقم 3، ولكن إذا شعرت أنّه قويّ، قد تختار الرقم 4 أو 5.** | | | | | | | |
| --- | --- | --- | --- | --- | --- | --- | --- |
|  | أرى أن هذا التحذير | | | | | | |
| **E1** | **يمكن تجاهله بسهولة** | 1 | 2 | 3 | 4 | 5 | **مُلفِت** |
| **E2** | **ليس مخيفا أبدًا** | 1 | 2 | 3 | 4 | 5 | **مخيف جدًا** |
| **E3** | **لم يضف لمعلوماتي** | 1 | 2 | 3 | 4 | 5 | **أضاف لمعلوماتي** |

| **يرجى إكمال هذا القسم إذا كنتم من غير المدخنين فقط** | | أعارض بشدة | أعارض | محايد | أوافق | أوافق بشدة |
| --- | --- | --- | --- | --- | --- | --- |
| **E4** | **عند رؤيتي لهذا التحذير** إزدادت **رغبتي** بأن أبقى غير مدخّن |  |  |  |  |  |
| **E5** | **أثّر بي هذا التحذير حتى** أصبحت **واثق** مِن أنّني لن أبدأ بالتدخين |  |  |  |  |  |

| **يرجى إكمال هذا القسم إذا كنتم من المدخنين فقط** | | أعارض بشدة | أعارض | محايد | أوافق | أوافق بشدة |
| --- | --- | --- | --- | --- | --- | --- |
| **E6** | **أثّر بي هذا التحذير حتى** أنه **يدفعني** إلى الإقلاع عن التدخين |  |  |  |  |  |
| **E7** | **أثّر بي هذا التحذير حتى** أني **واثق** مِن أنّني أستطيع الإقلاع عن التدخين |  |  |  |  |  |
| **E8** | **هل لديك ملاحظات أخرى على التحذير؟** | | | | | |

**معلومات شخصية:**

| **A1** | الجنس | 1 | ذكر |
| --- | --- | --- | --- |
|  |  | 2 | أنثى |
| **A2** | العمر |  | __________ سنة |
| **A3** | الوضع الإجتماعي | 1 | متزوج(ة) |
|  |  | 2 | أعزب / عزباء**🡨 A5** |
| **A4** | عدد الأبناء (ذكور وإناث) |  | __________ ولد |
| **A5** | المستوى التعليمي | 1 | ثانوية عامة |
|  |  | 2 | طالب كلية مجتمع |
|  |  | 3 | طالب جامعي |
|  |  | 4 | أنهى كلية مجتمع |
|  |  | 5 | أنهى الدراسة الجامعية |
| **A6** | التخصص | | |
