# Supplementary material for: Perceptions of young Jordanian adults to proposed anti-tobacco pictorial warning labels
Source: BMC Public Health. 2011 May 31;11:414. doi: 10.1186/1471-2458-11-414 (PMC3141444; doi:10.1186/1471-2458-11-414)
Supplement: Additional file 2 — Survey instrument - English version. This is a translation of the survey instrument that was utilized to collect data for this research. [file 1471-2458-11-414-S2.DOCX]

**Evaluation of the impact of pictorial warnings on cigarette packs in Jordan**

**Your attitudes and behavior in relation to tobacco:**

| **To what extent do you agree or disagree with the following statements.** | | Strongly Agree | Agree | Neutral | Disagree | Strongly Disagree |
| --- | --- | --- | --- | --- | --- | --- |
| **B1** | Smoking is harmful to smokers |  |  |  |  |  |
| **B2** | Exposure to cigarette smoke is harmful to smokers and non-smokers |  |  |  |  |  |

| **Please circle the most appropriate answer** | | | |
| --- | --- | --- | --- |
| **C1** | Is there currently any warning on the cigarette pack? | 0 | No **🡪 Skip to C4** |
|  |  | 1 | Yes |
| **C2** | Where is it located? | 1 | Front |
|  |  | 2 | Side |
|  |  | 3 | Back |
|  |  | 4 | Both front and back |
| **C3** | What does it say? | 1 | Smoking is addictive and can harm you |
|  |  | 2 | Smoking harms adults and children |
|  |  | 3 | Smoking causes serious and deadly illnesses |
|  |  | 4 | I don’t know |
| **C4** | Are you currently: | 1 | A Regular Cigarette Smoker |
|  |  | 2 | An Occasional Cigarette Smoker |
|  |  | 3 | Ex-smoker **🡪 Skip to D1** |
|  |  | 4 | A Non-Smoker **🡪 Skip to D1** |
| **C5** | Are you thinking of quitting in the coming 6 months? | 0 | No |
|  |  | 1 | Yes |
| **C6** | During the past 12 month, have you tried quitting smoking cigarettes? | 0 | No **🡪 Skip to D1** |
|  |  | 1 | Yes |
| **C7** | When was the attempt? | 1 | 6 months ago or less |
|  |  | 2 | More than 6 months ago |

**Reactions towards current pictorial**

| **D1** | **Have you ever seen this pictorial warning** | 0 | No |
| --- | --- | --- | --- |
|  |  | 1 | Yes |

| **Below there is a set of opposing adjectives. Please circle the number between the two adjectives that most describes your reaction to the warning you just saw. For example, if you felt that it was neither weak nor strong, you might circle the number '3', but if you felt that it was strong, you might circle the number '4' or '5'.** | | | | | | | |
| --- | --- | --- | --- | --- | --- | --- | --- |
|  | ***I thought this warning was*** | | | | | | |
| **D2** | **Not noticeable** | 1 | 2 | 3 | 4 | 5 | **Noticeable and attracts attention** |
| **D3** | **Not scary** | 1 | 2 | 3 | 4 | 5 | **Very scary** |
| **D4** | **Not informative** | 1 | 2 | 3 | 4 | 5 | **Informative and adds to my knowledge** |

| **Please complete this section ONLY if you are a Non-Smoker** | | Strongly Disagree | Disagree | Neutral | Agree | Strongly Agree |
| --- | --- | --- | --- | --- | --- | --- |
| **D5** | Seeing this warning motivates me to remain a nonsmoker |  |  |  |  |  |
| **D6** | After seeing this warning, I am confident that I will not start smoking |  |  |  |  |  |

| **Please complete this section ONLY if you are a Smoker** | | Strongly Disagree | Disagree | Neutral | Agree | Strongly Agree |
| --- | --- | --- | --- | --- | --- | --- |
| **D7** | Seeing this warning motivates me to try to quit smoking |  |  |  |  |  |
| **D8** | After seeing this warning, I am confident that I could quit smoking |  |  |  |  |  |

| **D9** | **Do you have any additional comments regarding this warning?** |
| --- | --- |

**Reactions towards proposed pictorial**

| **Below there is a set of opposing adjectives. Please circle the number between the two adjectives that most describes your reaction to the warning you just saw. For example, if you felt that it was neither weak nor strong, you might circle the number '3', but if you felt that it was strong, you might circle the number '4' or '5'.** | | | | | | | |
| --- | --- | --- | --- | --- | --- | --- | --- |
|  | ***I thought this warning was*** | | | | | | |
| **E1** | **Not noticeable** | 1 | 2 | 3 | 4 | 5 | **Noticeable and attracts attention** |
| **E2** | **Not scary** | 1 | 2 | 3 | 4 | 5 | **Very scary** |
| **E3** | **Not informative** | 1 | 2 | 3 | 4 | 5 | **Informative and adds to my knowledge** |

| **Please complete this section ONLY if you are a Non-Smoker** | | Strongly Disagree | Disagree | Neutral | Agree | Strongly Agree |
| --- | --- | --- | --- | --- | --- | --- |
| **E4** | Seeing this warning motivates me to remain a nonsmoker |  |  |  |  |  |
| **E5** | After seeing this warning, I am confident that I will not start smoking |  |  |  |  |  |

| **Please complete this section ONLY if you are a Smoker** | | Strongly Disagree | Disagree | Neutral | Agree | Strongly Agree |
| --- | --- | --- | --- | --- | --- | --- |
| **E6** | Seeing this warning motivates me to try to quit smoking |  |  |  |  |  |
| **E7** | After seeing this warning, I am confident that I could quit smoking |  |  |  |  |  |

| **E8** | **Do you have any additional comments regarding this warning?** |
| --- | --- |

**Personal Information**

| **A1** | You are | 1 | Male |
| --- | --- | --- | --- |
|  |  | 2 | Female |
| **A2** | How old are you? |  | _________ Years |
| **A3** | You are | 1 | Married |
|  |  | 2 | Single **🡪 Skip to A5** |
| **A4** | How many children do you have? |  | _________ Children |
| **A5** | What is the highest educational level you have completed? | 1 | High School Certificate |
|  |  | 2 | College student “diploma” |
|  |  | 3 | University student |
|  |  | 4 | Completed College “diploma” |
|  |  | 5 | Completed university |
| **A6** | Major |  | |
